# Influence of multiple stapler firings used for rectal division on colorectal anastomotic leak rate

**DOI:** 10.1007/s00464-017-5611-0

**Published:** 2017-06-20

**Authors:** Tamara Braunschmid, Nikolaus Hartig, Lukas Baumann, Bernhard Dauser, Friedrich Herbst

**Affiliations:** 1 Department of Surgery, Medical University of Vienna, Spitalgasse 23, 1090 Vienna, Austria; 2 Department of Surgery, St. John of God Hospital, Vienna, Austria; 3 Medical Faculty, Sigmund Freud University, Vienna, Austria; 4 Center for Medical Statistics, Informatics and Intelligent Systems, Medical University of Vienna, Vienna, Austria

**Keywords:** Multiple stapler firings, Anastomotic leakage, Compression anastomosis, Stapler anastomosis, Rectal division, Colorectal diseases

## Abstract

**Background:**

Anastomotic leakage following colorectal resection remains one of the most significant complications with relevant morbidity and mortality. There is evidence that a higher number of stapler firings for rectal division can affect the leak rate in double stapling anastomosis. However, there are no data concerning compression anastomosis. We present our institutional experience addressing this issue.

**Design:**

This is a retrospective review of a prospective institutional database of patients undergoing colonic and rectal resection for benign and malignant indications between January 2008 and December 2014 at the surgical department of the St. John of God Hospital, Vienna. Inclusion criteria were rectal division with linear stapling devices and construction of anastomosis to the rectal stump using a circular stapler or compression device.

**Results:**

Three hundred eighty two (196 female; 51.3%) patients were included. Mean age was 65.8 years (range: 18–95) Indications for the operation included diverticular disease (44.8%), colorectal carcinoma (51.6%), inflammatory bowel disease (1.8%), and adenoma (1.8%). A laparoscopic approach was employed in 334 cases (87.4%); in 170 patients (44.9%), a compression anastomosis was created. One, two, and three or more stapler cartridges were used for rectal division in 58.4, 33.5, and 8.1%, respectively. Male gender, neoadjuvant therapy, rectal cancer as an underlying disease, laparoscopic surgical approach, and duration of operation longer than 200 min are leading causes for the usage of more than one stapler cartridge. Overall leak rate was 4.7% (18/382). The only factor associated with the occurrence of leakage was the use of three or more stapler cartridges for the closure of the rectal stump (*p* = 0.002).

**Conclusion:**

Our data support that multiple stapler firings for rectal division following colorectal resection has a major impact on anastomotic leak rate. Especially in laparoscopic surgery efforts should be made to minimize the number of stapler cartridges used.

Anastomotic leakage represents one of the most severe complications after formation of a colorectal anastomosis, with relevant morbidity and mortality. In general, anastomotic leak rates range between 3.2 and 36% [1–9].

The basic requirements for anastomotic healing are an appropriate blood supply, healthy bowel ends, and a tension-free anastomosis [10–12].

Many studies have explored multiple variables in order to define their extent of influence on anastomotic leakage. Recent studies reported BMI to be an independent risk factor for the increase in anastomotic leak rate [13]. Besides, also the classification of the American Society of Anesthesiologists (ASA) seems to be an important factor because patients with higher ASA-scores also have a higher occurrence of anastomotic leaks [14]. Furthermore, the duration of operation, which often represents the complexity of an individual surgical procedure, seems to have an influence on anastomotic safety [3, 14]. Both, an inflamed bowel seen in acute diverticulitis or inflammatory bowel disease as well as previously irradiated bowel because of radio-chemotherapy can represent a difficult environment for the creation of a safe colorectal anastomosis [4, 15]. However, the most critical factor regarding anastomotic leaks is the level of the anastomosis, which seems to have a severe impact on the leakage rate, regardless of an open or laparoscopic approach [1–4, 16, 17]. Leak rates are higher following low anterior resection, no matter which technique was used [2, 16–18]. Particularly in laparoscopic colectomy, the narrow confines of the pelvis often results in an unideal cutting angle, leading to the use of more than one stapler cartridge for rectal division [2–4].

Several studies demonstrated the influence of multiple stapler firings on anastomotic leak rate [2–4]. However, there are no studies discussing the influence of multiple stapler firings on the safety of compression anastomoses.

The aim of the study is to present our institutional experience concerning anastomotic safety.

## Materials and methods

### Patient selection and surgical procedures

A retrospective analysis of a prospectively maintained institutional database of all colorectal resections, performed between November 2008 and December 2014, was carried out. We only included cases in which a colorectal anastomosis was performed after rectal transection with linear staplers. Anastomosis was created with a circular stapling or compression device (ColonRing^®^, NovoGI LTD., Netanya, Israel). Out of 827 patients gathered in the database, 382 patients fulfilled the inclusion criteria. The indications for an operation included colorectal carcinoma or adenoma, inflammatory bowel disease, or diverticular disease. Whenever a diverting stoma was planned, patients received preoperative bowel preparation with three liters of polyethylene glycol solution on the day before the operation. The remaining patients received a rectal enema only. The BMI was defined according to the definitions of the World Health Organization (WHO) [19, 20].

All anastomoses were created intracorporeally. The closure of the rectal stump was performed using one or more firings of a linear stapling device. Thereafter, colorectal anastomosis was done using a circular stapling device or a compression anastomotic device. The anastomotic integrity was checked by the inspection of the doughnuts and insufflation of air.

From January 2009 onwards, for construction of rectal anastomosis, a compression anastomotic device was used exclusively by two members of staff. This was part of a prospective evaluation of this new technology and was registered with https://ClinicalTrials.gov under NCT01056913.

Anastomotic leakage was defined as proposed by Rahbari et al. [8]. A computed tomography was not done routinely in patients that received an anastomosis. Nevertheless, if there were any clinical suspicions of a leakage, a computed tomography was done to verify or falsify the assumptions.

The primary endpoint of this retrospective survey was the occurrence of an anastomotic leak.

### Statistical analyses

The statistical analysis was performed using R version 3.3.0. All variables were categorized. The univariate associations were tested with Chi-Square Tests or—if assumptions were not met—Fisher’s Exact Tests. As this is an exploratory study we did not correct for multiple testing. *p* < 0.05 was considered significant for all tests. To analyze the multivariate impact of independent variables on the number of stapler cartridges used, a multivariate logistic regression model was conducted. Therefore, the number of cartridges was dichotomized (1 vs. 2 or more cartridges). All variables were included in the first model, and a stepwise selection with the Akaike Information Criterion (AIC) was performed to find the most influential variables. A multivariate model for the risk of anastomotic leakage was planned but as only one variable showed a univariate association, this model was not computed.

### Institutional review board

The study protocol was approved by the local ethics committee.

## Results

The study population of 382 patients included 186 (48.7%) men and 196 (51.3%) women. Mean age at operation was 65.8 years (range: 18–95 years). Fifty point five percent of the patients were older than 65 years. The mean Body Mass Index (BMI) within the cohort was 25.91 (range 15.1–55.4). Demographic data are shown in Table 1.

**Table 1:** Demographic data; *n* = 382

Variable	*n* (%)
Sex	
Male	186 (48.7)
Female	196 (51.3)
BMI	
Underweight	11 (2.9)
Normal	150 (39.3)
Overweight	155 (40.6)
Obesity	66 (17.3)
ASA	
I	209 (54.7)
II	121 (31.7)
III	48 (12.6)
IV	4 (1.0)
Age at surgery (years)	
<50	47 (12.3)
50–64.9	136 (35.6)
65–74.9	126 (33.0)
≥75	73 (19.1)
Diagnosis	
Rectal cancer	97 (25.4)
Colon cancer	99 (25.9)
Adenoma	7 (1.8)
Diverticular disease	172 (45.0)
Inflammatory bowel disease	7 (1.8)
Surgical approach	
Laparoscopically	334 (87.4)
Open	48 (12.6)
Type of operation	
Sigmoid resection	166 (43.5)
Left hemicolectomy	74 (19.4)
Anterior resection	57 (14.9)
Low anterior resection	85 (22.3)

Influence of multiple stapler firings used for rectal division on colorectal anastomotic leak rate

### Indications for operation

Fifty-one point six percent of patients underwent resection for colorectal cancer, whereas 48.4% were treated because of a benign indication such as adenoma (1.8%), diverticular (44.8%) or inflammatory bowel disease (1.8%).

Compared to benign indications, in cancer operations more often three or more stapler cartridges were used for the closure of the rectal stump (13.7 vs. 2.2%; *p* < 0.001), anastomoses were constructed more frequently in the lower third of the rectum (39.6 vs. 2.7%; *p* < 0.001) and a protective stoma was created more often (33.5 vs. 2.7%; *p* < 0.001).

Out of 97 cases with rectal cancer, 47 patients (48.5%) received neoadjuvant chemoradiation (*n* = 22), short-course radiotherapy (25 Gy; *n* = 20), or chemotherapy only (*n* = 5).

### Surgical procedures and related complications

The majority of procedures (87.4%) were performed laparoscopically. In 5.2%, a conversion to the open technique was required. A primary open access was only used in patients who had already had multiple open abdominal surgeries. In 67.1% (57/85) of the patients who underwent a low anterior resection, a protective ileostomy was created.

Forty-three point five percent of patients underwent a sigmoid resection, 19.4% received a left hemicolectomy, in 14.9% a high anterior resection, and in 22.3% a low anterior resection was performed. In 225 cases (58.9%), the anastomosis was located in the upper third of the rectum (12–16 cm), in 74 (19.4%) it was located in the middle third (7–11 cm), and in 83 (21.7%) it was located in the lower third (6 cm or lower). In 68.7% of all patients with anastomosis in the lower third, a diverting loop ileostomy was created.

In 58.4% (*n* = 223) of all resections only one stapler cartridge was used to divide the rectum, whereas in 33.5% (*n* = 128) two magazines were used and in 8.1% (*n* = 31) three or more magazines were required.

For the construction of the anastomosis, a circular stapler was used in 55.5% (*n* = 212), whereas 44.5% (*n* = 170) were performed with a compression anastomotic device. Within our cohort patients who received an anastomosis in the lower third (22.3%) showed a significantly higher anastomotic leak rate without a difference between stapled and compression anastomosis. In general, patients who received a compression anastomosis had significantly lower anastomoses (*p* < 0.001) and therefore a higher protective stoma rate (*p* < 0.001).

All stomata were created as protective stomata. Three patients hat no reoperation because of an intermittently risen operation-risk or because of their individual decision to keep the stoma.

The average duration of the operation was 209 min (range: 45–630 min). Within laparoscopic procedures the mean operation duration was 200 min (range: 45–630 min), whereas within interventions in open technique, the mean operation duration was 194 min (range: 85–490 min).

The mean hospital stay was 9 days (range: 2–124 days). There was a significant difference within the in-hospital stay regarding the surgical approach. Patients who underwent an open surgical procedure had significantly longer postoperative hospital stays than patients that received a laparoscopic procedure (13 vs. 8 days; *p* = 0.001). As expected, the development of an anastomotic leak led to a statistically significant prolongation of the hospital stay (27.5 days with vs. 8 days without leak; *p* < 0.001).

### Preconditions affecting the number of stapler firings

Within the univariate analysis—see Table 2, we found several factors that could possibly have an influence on the number of linear stapler firings used for rectal division. From the patients’ preconditions gender, previous diagnosis and neoadjuvant therapy showed significant results. Regarding the surgical procedure itself, we found significant differences for the type of surgical approach, the type of operation, the level of anastomosis, the anastomotic technique, as well as for the duration of the operation. Multivariate analysis showed that male gender, rectal cancer as an underlying disease, neoadjuvant therapy, laparoscopic approach, and duration of operation longer than 200 min have the most relevant influence on the usage of more than one linear stapler magazine to divide the rectum.

**Table 2:** Patient characteristics and number of stapler cartridges used for rectal transection

Variable	Total no. of patients	Number of stapler cartridges			*p* value	Multivariate analysis	
	*n* = 382	1 *n* = 223 (%)	2 *n* = 128 (%)	≥3 *n* = 31 (%)		Odds ratio	CI (95%)
Sex							
Male	186	96 (51.6)	68 (36.6)	22 (11.8)	**0.007**	*1.00*	–
Female	196	127 (64.8)	60 (30.6)	9 (4.6)		0.69	0.43–1.11
BMI							
Underweight	11	7 (63.6)	4 (36.4)	0	0.957		
Normal	150	89 (59.3)	51 (34.0)	10 (6.7)			
Overweight	155	88 (56.8)	52 (33.5)	15 (9.7)			
Obese	66	39 (59.1)	21 (31.8)	6 (9.1)			
ASA							
I	209	121 (57.9)	71 (34.0)	17 (8.1)	0.627		
II	121	72 (59.5)	41 (33.9)	8 (6.6)			
III	48	29 (60.4)	14 (29.2)	5 (10.4)			
IV	4	1 (25.0)	2 (50.0)	1 (25.0)			
Age at surgery (years)							
<50	47	27 (57.4)	16 (34.0)	4 (8.5)	0.727		
50–64.9	136	74 (54.4)	48 (35.3)	14 (10.3)			
65–74.9	126	81 (64.3)	37 (29.4)	8 (6.3)			
≥75	73	41 (56.2)	27 (37.0)	5 (6.8)			
Underlying diagnosis							
Benign	185	127 (68.6)	54 (29.2)	4 (2.2)	**<0.001**		
Malignant	197	96 (48.7)	74 (57.8)	27 (13.7)			
Diagnosis							
Rectal cancer	97	24 (24.7)	50 (51.5)	23 (23.7)	**<0.001**	*1.00*	–
Colon cancer	99	71 (71.7)	24 (24.2)	4 (4.0)		0.19	0.09–0.38
Adenoma	7	3 (42.9)	4 (57.1)	0		0.86	0.16–5.16
Diverticular disease	172	119 (69.2)	49 (28.5)	4 (2.3)		0.20	0.10–0.39
IBD	7	6 (85.7)	1 (14.3)	0		0.06	0.00–0.39
Previous therapy							
No	335	212 (63.3)	109 (32.5)	14 (4.2)	**<0.001**	1.00	–
Yes	47	11 (23.4)	19 (40.4)	17 (36.3)		*2.36*	0.93–6.37
Surgical approach							
Laparoscopic	334	181 (54.2)	123 (36.8)	30 (9.0)	**<0.001**	*1.00*	–
Open	48	42 (87.5)	5 (10.4)	1 (8.1)		0.11	0.03–0.27
Type of operation							
Sigmoid resection	166	123 (74.1)	41 (24.7)	2 (1.2)	**<0.001**		
Left hemicolectomy	74	48 (64.9)	23 (31.3)	3 (4.1			
Anterior resection	57	30 (52.6)	22 (38.6)	5 (8.8)			
Low anterior resection	85	22 (25.9)	42 (24.7)	21 (24.7)			
Anastomotic device							
Stapler	212	140 (66.0)	63 (29.7)	9 (4.2)	**<0.001**		
Compression	170	83 (48.8)	65 (38.2)	22 (12.9)			
Anastomotic height							
Low (≤6 cm)	83	23 (27.7)	40 (48.2)	20 (24.1)	**<0.001**		
Middle (>6–12 cm)	74	41 (55.4)	27 (36.5)	6 (8.1)			
High (>12–16 cm)	225	159 (70.7)	61 (27.1)	5 (2.2)			
Duration of operation							
≤200 min	196	134 (68.4)	54 (27.6)	8 (4.1)	**<0.001**	1.00	–
>200 min	186	89 (47.8)	74 (39.8)	23 (12.4)		*1.92*	1.20–3.10

Statistically significant *p* values are given in bold

Highest values are given in italic

Influence of multiple stapler firings used for rectal division on colorectal anastomotic leak rate

### Anastomotic leakage

Eighteen of the 382 patients (5.4%, male *n* = 10) developed an anastomotic leak—see Table 3. Those patients’ mean age was 61.5 years (range: 31–79 years), the mean BMI was 26.9 (range: 19.1–55.4), the mean duration of the surgical intervention was 245 min (range: 141–490 min), and the mean postoperative in-hospital stay was 39.6 days (range: 9–115 days). In 7 patients (38.9%), a stapling device was used to create the anastomosis, whereas 11 times (61.1%) a compression device was utilized. To divide the rectum in 6 cases (33.3%), one stapler cartridge was used, in 6 cases (33.3%) two stapler cartridges were used, and in 6 cases (33.3%), three up to six stapler cartridges were needed.

**Table 3:** Patient characteristics and anastomotic leak rate

Variable	Patients with anastomotic leak	Leakage (%)	*p* value
Global leak rate	18/382	4.7	–
Sex			
Male	10/186	5.4	0.722
Female	8/196	4.1	
BMI			
Underweight	0/11	–	0.970
Normal	8/150	5.3	
Overweight	7/155	4.5	
Obese	3/66	4.6	
ASA			
I	7/209	3.4	0.100
II	6/121	4.9	
III	4/48	8.3	
IV	1/4	25.0	
Age at surgery (years)			
<50	3/47	6.4	0.894
50–64.9	7/136	5.2	
65–74.9	5/126	3.9	
≥75	3/73	4.1	
Underlying diagnosis			
Benign	5/185	2.7	0.120
Malignant	13/197	6.6	
Diagnosis			
Rectal cancer	8/97	8.3	0.377
Colon cancer	5/99	5.1	
Adenoma	0/7	–	
Diverticular disease	5/172	2.9	
IBD	0/7	–	
Neoadjuvant therapy			
No	13/335	3.9	0.057
Yes	5/47	10.6	
Surgical approach			
Laparoscopic	16/334	3.9	1.000
Open	2/48	4.1	
Type of operation			
Sigmoid resection	7/166	4.2	0.121
Left hemicolectomy	1/74	1.4	
Anterior resection	2/57	3.5	
Low anterior resection	8/85	9.4	
Number of cartridges			
1	6/223	2.7	**0.002**
2	6/128	4.7	
≥3	6/31	19.4	
Anastomotic device			
Stapler	7/212	3.3	0.226
Compression	11/170	6.5	
Anastomotic height			
Low (≤6 cm)	7/83	8.4	0.204
Middle (>6–12 cm)	2/74	2.7	
High (>12–16 cm)	9/225	4.0	
Duration of operation			
≤200 min	8/196	4.1	0.722
>200 min	10/186	5.4	

Statistically significant *p* value is given in bold

Influence of multiple stapler firings used for rectal division on colorectal anastomotic leak rate

No patient in this group died. Regarding treatment of leaks only four anastomoses (22.2%) could not be salvaged (2 open and 2 laparoscopic Hartmann’s procedures). Eight patients underwent an abdominal revision with the creation of an ileostomy (4 open, 4 laparoscopic), and one underwent laparotomy with redo of the anastomosis (ileostomy already present). Three patients received a transanal endoscopic vacuum therapy (one needed additional laparotomy and lavage for persistent peritonitis). Two patients with ultralow compression anastomosis and a protective ileostomy already in place underwent a transanal removal of the device and repair of the anastomosis.

All salvaged anastomoses healed and the stomata were closed at a later stage. Only one patient in the Hartmann group did not have the terminal colostomy reversed.

Interestingly, within univariate analysis, we could not find any patient-related risk factors such as age, BMI, or ASA (*p* = 0.970, *p* = 0.100) having a significant influence on the leak rate. Not even technical factors such as surgical approach, type of operation, anastomotic height, or anastomotic technique seemed to have an influence on anastomotic safety (*p* = 1.000, *p* = 0.121, *p* = 0.204, *p* = 0.226). The only variable showing a significant influence on the development of an anastomotic leak was the number of linear stapler firings that have been used for rectal division (*p* = 0.002).

## Discussion

Anastomotic leakage remains one of the most serious complications after colorectal resection [8, 21]. It increases morbidity and mortality as well as reoperation rate and hospital stay [7, 21]. In the past, many studies have tried to identify potential risk factors that favor the development of an anastomotic leak. [1–4, 14, 17].

Many risk factors were suspected to have an appreciable influence on the occurrence of anastomotic leaks. Often patients’ preconditions such as age, gender, BMI, or ASA have been assumed to cause a higher risk for the integrity of an anastomosis [13, 14, 18, 22]. However, in our cohort, we were not able to demonstrate a correlation between patient characteristics and leak rate. Regarding literature, there is evidence that technical aspects have an even bigger influence on anastomotic safety than patient preconditions [1, 14]. The anastomotic level as well as the number of linear stapler firings that are used for rectal division seems to have a major impact on the development of an anastomotic dehiscence [2–4, 16]. Previous studies have demonstrated the significance of the anastomotic level as an independent risk factor for anastomotic safety. [1, 3, 4]. Anyhow within our cohort, we could not confirm these findings.

Several studies have discussed the relationship between the number of stapler firings and the rate of anastomotic leaks. In 2008, Ito et al. demonstrated a significant relationship between the number of stapler cartridges used for rectal division and the occurrence of anastomotic leaks [2]. One year later, Kim and colleagues were able to validate these findings by their prospective study including 270 patients [3]. This report gave strong evidence that the application of multiple stapler cartridges and an anastomosis located in the middle or lower rectum are major contributors for development of an anastomotic leak [3]. Park et al. could also verify these findings in 2013 [4].

In our study, the number of stapler firings emerged to have the most powerful influence on the occurrence of anastomotic leaks. In contrast to many other studies, anastomotic level was not significant. There may be two explanations for this discrepancy; on one hand three or more stapler magazines were predominantly used in the lower third, on the other hand the number of patients in our series could be too small.

There are several reasons as to why more than one stapler cartridge is needed to divide the large rectum. Firstly, the large diameter of the wall in the middle and lower rectum could be the reason to use more than one magazine [1–3]. Secondly, if initially a small magazine with 45 mm instead of 60 mm is used, there is a higher chance that in the end more than one magazine has to be applied to divert the large bowel [1]. Thirdly, the limited space and angle within the laparoscopic surgical approach as well as a narrow male pelvis is a risk factor that often leads to the usage of two or more magazines [1–3].

Also our study confirms these findings. Multivariate analysis showed that besides male gender also rectal cancer as an underlying disease as well as neoadjuvant therapy and laparoscopic procedure seemed to be a risk factor that more stapler cartridges are used.

In literature, it has been demonstrated that more stapler cartridges are also used in patients with a longer operation time [3]. Again, we could show this effect within our data. The usage of more stapler firings to divide the bowel is more likely in surgical interventions that are more complex and taking longer than 200 min.

The viability of the bowel ends to be connected is an essential factor for anastomotic safety. In case of preoperative chemoradiation or in the event of inflammation of the bowel wall, the integrity of the tissue is not always guaranteed. For diverticulitis, the laparoscopic approach seems to have a positive effect concerning the risk of anastomotic leak [23]. Interestingly, in our study, we found a significantly lower stoma rate in patients with benign primary disease which seems to originate in the fact that patients with benign diseases have significantly higher anastomoses than patients with a malignant underlying disease.

According to the literature, anastomoses to the middle or low rectum are associated with a higher risk for anastomotic leakage [1, 3]. As a consequence, the creation of a protecting stoma should be considered in high-risk patients particularly when they receive a very low anastomosis [1, 3, 4, 14, 24, 25].

Laparoscopic colectomy was first described in the literature in 1991 by Jacobs and colleagues [26]. Following this publication, numerous studies have evaluated the safety and feasibility of laparoscopic versus the open technique for colorectal resections under many different conditions. The most crucial points concerning laparoscopic colorectal resection were on the one hand the adequacy of the cancer operation in regard to free tumor margins and sufficient lymph node resection, and on the other hand the feasibility and safety for sphincter-preserving surgery of the low and middle rectum [25]. Many studies in the last two decades demonstrated that laparoscopic colorectal resections and open resections have comparable oncological and functional results [27–30]. The leak rates are similar, even in patients that underwent neoadjuvant radiotherapy [27]; the in-hospital stay was even shorter in laparoscopically operated patients [31, 32]. Our data confirm that a laparoscopic approach is neither followed by a higher leak rate nor by a longer in-hospital stay; in contrast, patients that underwent a laparoscopic surgical procedure had shorter postoperative in-hospital stays than those with an open surgical approach.

Comparing compression to stapled anastomoses, there were no significant differences regarding duration of operation, postoperative hospital stay, or anastomotic leak rate.

## Conclusion

The occurrence of anastomotic leaks following colorectal resection is still one of the most serious complications. Our experience shows that patients’ preconditions are not the most determining variables concerning anastomotic leaks. On the contrary, technical details within the operation have a decisive impact on anastomotic leakage. It can be said that the most important factor for anastomotic safety is the use of fewer stapler cartridges in colorectal anastomosis. If it is foreseeable that more than one stapler cartridge will be required for rectal division, the surgical approach should be reevaluated. Be it the placement of another trocar or a small laparotomy to finish the rectal division using a hand port, regardless, an adjustment to the surgical approach should be considered in order to minimize the number of stapler cartridges used.
